# Metacognition in musical practices: two studies with beginner and expert Brazilian musicians

**DOI:** 10.3389/fpsyg.2024.1331988

**Published:** 2024-02-22

**Authors:** Rosane Cardoso de Araújo, Rafael Stefanichen Ferronato, Flávio Denis Dias Veloso

**Affiliations:** ^1^Department of Arts, Federal University of Parana, Curitiba, Brazil; ^2^The Brazilian National Council for Scientific and Technological Development (CNPq), Brasília, Brazil; ^3^School of Fine Arts, Pontifical Catholic University of Parana, Curitiba, Brazil

**Keywords:** metacognition, metacognitive process, musical practices, beginners musicians, expert musicians

## Abstract

Metacognition is essential in the musical learning process as it involves understanding the purpose of each task, its planning, execution and evaluation. Considering the relevance of metacognitive processes, our objective in this study was to investigate how expert and beginner musicians manifest and verbalize their metacognitive processes in the context of preparing repertoire for a performance. The method used was a multi-case study carried out in two different contexts: with the five members of a brass quintet made up of professional musicians and with three beginner university violin students. The results obtained indicated that even at different levels of expertise, metacognitive processes were present in the musical practices of participants in the two contexts investigated. It was found that in both cases time management is a component of the preparation process in metacognitive regulation, however, for the beginner violinists in our sample it was a significantly more complex task than for the professional brass players. Regarding the learning monitoring and evaluation processes, it was possible to verify that beginner instrumentalists as well as professional musicians used declarative, conditional and procedural knowledge to carry out and reflect on their musical practices. These results have implications for both the individual and collective study process and for teaching processes. It is also observed that reflective thinking must accompany the processes of individual and collective interpretative-musical practices, considering that the musical results desired by musicians are related to the quality of cognitive, behavioral, affective and motivational undertakings pertinent to control and regulation of metacognitive processes.

## 1 Introduction

Metacognition occupies a prominent position among the factors involved in learning processes from the perspective of cognitive and developmental psychology (Flavell et al., 2002). According to Flavell (1979) to master metacognitive skills is to understand the purpose of each task, plan how to complete it, consciously apply and change study strategies and evaluate the results of performance and of the learning outcome. Ribeiro (2003) explains that metacognition is higher-order cognition, which guides thinking about thinking, knowledge about one’s own knowledge and the self-regulation of cognitive processes.

Anderson et al. (2001) defined metacognitive knowledge as awareness and knowledge of one’s own cognition, which encompasses strategic knowledge, knowledge about cognitive tasks, including contextual and conditional knowledge, as well as self-knowledge. Thinking about thinking, learning how to learn, cognition about cognition, goal management, coordination and monitoring of mental activities are components of metacognition explored by various authors and indicate that this construct, due to its complexity, is understood as a set of processes (Jacobs and Paris, 1987; Paris and Winograd, 1990; Schraw and Moshman, 1995; Kuhn, 2000; Ribeiro, 2003; Joly, 2007; Noushad, 2008; Chick et al., 2009; Benton, 2014; Bustos et al., 2014; Jordan, 2014; Bártolo-Ribeiro et al., 2016; Ozturk, 2017; Varga, 2017). Metacognitive processes allow musicians to engage in the planning and organization of instrumental practice tasks, the monitoring and evaluation of performances and the promotion of self-directed changes.

According to Flavell (1999), metacognitive regulation operates through three central processes: *planning*, *monitoring* and *evaluation*. These processes are manifested in a dynamic and non-linear way, allowing that, due to cognitive flexibility, elements of the three processes can be explored and combined at different times during a musical learning activity. *Planning* includes, through anticipatory thinking, outlining tasks, setting goals, mobilizing strategies and resources, and estimating the amount of time and effort invested. According to Portilho (2011), people generally develop a plan to execute a task when faced with a problem or a new situation. This organization will guide cognitive activity. *Monitoring* takes place through constant self-observation and allows the objectives and strategies to be revised so that the individual can achieve the stipulated goals. During monitoring, metacognitive knowledge reveals learners’ levels of awareness of their own functioning, manifesting itself in three forms: (i) “declarative knowledge,” which is the students’ understanding of what they know in terms of information, skills, strategies and resources; (ii) “conditional knowledge,” which is the discernment of when to mobilize a certain practice strategy; and (iii) “procedural knowledge,” which is the understanding of how to perform a task using specific procedures (Portilho, 2011). Finally, the *evaluation* involves judging their own behavior, verifying the quantity and quality of progress made, as well as the relevance of the resources and strategies used.

There are also recent theoretical models of metacognition, for example, the studies developed by; Drigas and Mitsea (2020a,b). The authors present a holistic and muti-scientific approach to identify the basic pillars of metacognition. They refer to eight fundamental pillars: (1) Deep theorical knowledge on our cognition; (2) Operation knowledge about the functionality of cognitive abilities; (3) self-monitoring; (4) self-regulation; (5) Adaptation; (6) Recognition; (7) Discrimination; (8)Mnemosyne. The authors argue that all the pillars are interdependent and function with some degree of autonomy, since that any improvement or malfunction in each pillar can affect the metacognitive mechanism as a whole. They also propose the use of a mindfulness model, which includes strategies to develop metacognitive skills, which increase the level of self-organization and awareness of individuals.

Beginner and expert musicians explore and improve metacognitive skills by reflecting on their study processes from the moment before direct practice, during the study and when verifying the results obtained. This process influences the quality of learning and motivation in individual and collective musical achievements. These findings reflect those of previous studies examining the relationship between musical learning and metacognition (Hallam, 2001; Jørgensen and Hallam, 2016; Ordóñez, 2016; Power and Powell, 2018; Concina, 2019; Veloso and Araujo, 2019). Hallam (2001) carried out a comparative study on the development of metacognitive skills between 22 professional musicians and 55 novices (aged 6–18), during the preparation of a repertoire. She found that professional musicians had well-developed metacognitive skills to identify their strengths and weaknesses, evaluate tasks and identify strategies to optimize performance, while novice students had less developed strategies that did not always optimize performance. The focus of our research, as well as in the investigation by Hallam (2001), was the study of the metacognitive processes of professional and beginner musicians, in musical practice and learning. Our sample, however, although limited by its small size in relation to Halam’s study, was original in carrying out the research in the Brazilian context, with a group of professional musicians, members of a brass quintet (*n* = 5) and a group of university students who were beginning to study the violin (*n* = 3). We do not seek to replicate the relevant study carried out by Hallam (2001), but we seek to follow our own methodology, to later contribute to the results already achieved by this author. The general objective of our study was to investigate how profissional musicians and beginner college musicians manifest and verbalize their metacognitive processes in the context of studying and preparing of a musical repertoire. Here, we present the methodological procedures applied to each case study, the results of the two studies (separately), and the discussion of the results cross-sectionally.

## 2 Method

The method used was a multi-case study, carried out in two different contexts: (i) with the five members of a brass quintet made up of professional musicians and (ii) with three beginner college violin students. The data was collected in Brazil, in the city of Curitiba (southern Brazil).

### 2.1 Participants

The first case study (Case 1) we present was carried out in 2022, with a brass quintet (two trumpet players, a horn player, a euphonium/trombonist and a tuba player - all men). All were professional and experienced instrumentalists, as they all had undergraduate and postgraduate degrees in music and worked as teachers and performers. They had been playing together regularly since 2017. The musicians’ ages ranged from 30 to 50 years old.

The second case study (Case 2) was carried out between 2018 and 2019 with three beginner university violin students (one woman and two men), aged between 19 and 22 years old. The three participants in this second case study were university students. They had never studied violin. One student was a music student and played electric guitar. The other two studied computer science and had no formal musical knowledge. All participants had small paid jobs outside the university.

### 2.2 Procedures

Data from case 1 come from the systematic observation of two trials and two semi-structured interviews in focus groups (conducted in Portuguese). The interview questions were developed based on the theoretical model of metacognitive regulation.^1^ This model includes the stages of planning (involving task selection, time organization, effort forecasting and outlining practice goals and strategies), monitoring (with emphasis on metacognitive experiences and knowledge), evaluation and self-reaction (involving domain parameters, performance standards, causal attributions and changes in the course of actions) (Flavell, 1979; Schraw and Moshman, 1995).

The data for Case 2 were collected through observation of the beginner college violin students in the classroom context, because we understood that the relationship with the teacher could have a significant influence on the students’ learning and metacognition, since they were all beginners on the violin. The study included class observations and semi-structured interviews with students. The interview guide was based on the Motivated Strategies for Learning Questionnaire (MSLQ), developed by Pintrich (1991).^2^ Pintrich et al. (2000) highlights in his MSLQ model: (a) students’ knowledge of general strategies for learning to think, (b) how, when and why to use different strategies, and (c) what is the relationship between cognitive components and motivational of each individual. Thus, the interviews used in case study 2 had two main sections: (1) the first section, which included questions about students’ motivation and expectations, their beliefs about their abilities to succeed in studying the violin, (2) and the second section included questions about the metacognitive process - planning, monitoring and evaluation - to verify learning strategies, cognitive and metacognitive skills. In this section, the questions were divided into 3 parts: about the participants’ study process, about their knowledge of the content covered in violin classes and about seeking help to assist their study with the teacher and classmates.

The interviews in both case studies were audio recorded and transcribed in full. Case observations were carried out: (1) through video recording of professional musicians’ rehearsals; and through notes in an “observation notebook” of beginner college violin students. The analysis of the material includes the content analysis steps identified by Bardin (2011): pre-analysis; categorization; treatment of results, inferences and interpretation. Analysis categories were defined *a priori* - Planning, Monitoring and Evaluation – and were selected from the data through semantic proximity (Bardin, 2011).

## 3 Results

### 3.1 First study: professional musicians from a brass quintet

During the data collection period, the ensemble was preparing for a recital. The program included the following musical pieces: “Go!,” by Anthony DiLorenzo; “Largo,” from Symphony n. 9, by A. Dvořák, “Bachianas Brasileiras n. 5,” by H. Villa-Lobos, “Spiritual Waltz,” by Enrique Crespo, “bRUMBA!,” by James M. Stephenson, “Fire Dance,” by Anthony DiLorenzo and “The Knight of the Hill” (1st mov.), by Giancarlo C. D’addona. The quintet performed a weekly rehearsal of approximately 2 hours. The dynamics of the rehearsals involved an initial collective warm-up session, a repertoire practice session – in which different practice strategies were mobilized, with an emphasis on part-whole strategies (Jørgensen and Hallam, 2016) and recital simulation – and a session closing, when individual reflections, perceptions and evaluations were shared in the group. In data collection, systematic observations of two rehearsals were carried out respecting the ensemble’s regular schedule; the two semi-structured focus group interviews were carried out in person immediately after the rehearsals.

In the interviews, when asked about the dynamics of rehearsals, the musicians highlighted the adoption of three main strategies: organizing the practice in line with the group’s artistic programming, the management of practice time considering the distribution of warm-up tasks and repertoire practice throughout the rehearsal, and the complexity of the musical material they were working on (giving more rehearsal time to challenging repertoires for the group). In this regard, the three properties of the goals cited by Schunk (2001, 2014) were explored: specificity, proximity and difficulty (see Table 1).

**Table 1:** Property of goals.

Property of goals	Result of interviews
Specificity: knowledge about the specific demands of the tasks performed	*(...)This end of the “E” section (pio mosso), for example, if we manage to maintain the tempo, maybe even reduce the dynamics so that we can maintain the tempo [singing the excerpt]* (Trumpeter player 1; rehearsal n. 1).
Proximity: short and medium term temporal delimitation	*(...) we will, in addition to repeating the recital in its entirety (in this year 2022), dedicate at least 50% of the rehearsal time to the new song* (Trumpet player 1, interview 1).
Difficulty – awareness about the complexity of goals and tasks	*(...) we went to the most difficult piece, the thorniest piece, and there we stayed for 80% of the rehearsal [expressions of agreement in the group]* (Trumpet player 1, interview 1).

Planning musical practice requires setting goals, which is understood as what you want to achieve in a conscious and intentional way. The data regarding the selection of tasks and the delineation of goals for the quintet’s rehearsals revealed the group’s consolidated experience, both in carrying out the warm-up exercises and in practicing the repertoire.

In the observations of the group’s rehearsals, it was possible to collect data on the instrumentalists’ initial contact with the rehearsal space, the first dialogs between the musicians, the warm-up session (the initial moment of the rehearsal) and the repertoire practice session (the central moment of the rehearsal). According to Portilho (2011), when a new situation or problem arises, people organize a plan to regulate the execution of the task. Thus, planning activities requires flexibility, considering the challenges that may require redirecting objectives and tasks during the rehearsal. Specifically regarding the *planning* of the repertoire, the following factors could be verified:

- Consideration of interpretative-musical and technical-instrumental challenges as criteria for selecting the repertoire: *“(...) when we chose to be part of this group, we also set ourselves challenges, we did not want to go out and do more of the same”* (Trumpet player 1, interview 1).
- The need for development and improvement of skills: *“We really wanted pieces that would make us grow as musicians, and that’s what happened”* (Trumpet player 1, interview 1). “*We have played Bachianas Brasileiras n.5 since 2016 until today in all rehearsals... and we still manage to find some issues to improve*” (Trumpet player 1, interview 1).

*Monitoring* consists of deliberate observation of the performance of musical practice activities. In this study, the monitoring of the brass quintet musicians was verified based on the three main types of knowledge that integrate metacognitive processes: declarative knowledge, procedural knowledge and conditional knowledge. Monitoring strategies based on metacognitive knowledge were verified by observing dialogs and verbalizations carried out during rehearsals and reports offered by musicians during collective interviews (see Table 2).

**Table 2:** Examples of types of metacognitive knowledge.

Types of knowledge (Flavell et al., 2002)	Data collected in the case study
Declarative: individuals’ knowledge about what they know and the strategies and personal resources they have at their disposal.	The mobilization of declarative metacognitive knowledge was verified in processes related to auditory-musical perception and social and musical interactions between musicians and also in the choice of repertoire: e.g.*“(...) choosing [the repertoire] involves appreciating the pieces, and when I hear something... I imagine the sound of the horn player, on the trumpet the sound of the trumpeter... and then I think about the sound”* (Euphonist, interview 2).
Procedural: awareness of how to perform a given task and which strategies and resources should be used	Procedural knowledge was verified especially during the participants’ reports on knowledge and strategies explored in solving problems during the construction of the performance: e.g. “*In the sentence they do in measure 22, we could breathe there”* (Trumpet player 1, rehearsal n.2)
Conditional: notion of when, where and for what reasons to use certain learning strategies	Data relating to conditional knowledge were identified in the dialogs during the practice sessions. Musicians reveal an understanding of technical and interpretative difficulties, cognitive demands (e.g. memorization) and physical effort requirements. “*In the phrase (…) in measure 22, we could breathe there*” (Trumpet player 1, rehearsal n.2)

In addition to metacognitive knowledge, another phenomenon deserves to be highlighted: metacognitive experiences, that is, conscious perceptions associated with performing a task (Flavell, 1979; Schraw and Moshman, 1995; Ribeiro, 2003; Veloso and Araujo, 2019). Data collected from observing the rehearsals suggested that the metacognitive strategies used by the musicians during collective practice included self-questioning (thinking out loud) *“I think my [G] is low”* (Trumpet player 1, rehearsal 1) and with evaluative inferences, *“I was really making a mistake!,” “(...) yeah, now it’s [good] [expressions of agreement in the group]*” (Trumpet player 1, rehearsal 1), *“He was! Now it was. Now it’s fitting!”* (Trumpet player 2, rehearsal 2).

Data from the interviews made it possible to relate aspects of individual study and collective study in overcoming challenges based on the verbalization of the strategies employed and personal perceptions of performance (Ribeiro, 2003) *- “So we practice at home, and how many times have we said that, this phrase here, right: ‘gee, but I practice at home and at home everything works out’ [expressions of agreement in the group]”* (Trumpet player 1, interview 1). The sense of collective achievement was another aspect related to metacognitive experiences, particularly in situations where the musicians realized that they had overcome a challenge: *“That was an achievement! We were overjoyed! [Expressions of agreement in the group]”* (“Trumpet player 1,” interview 1).

In the *evaluation*, the expression of opinions about individual and collective performance was frequently observed throughout the rehearsals. Assessments were based, among other factors, on comparative parameters, considering data from the group’s performance history: *“It was the best time we have done so far [expressions of agreement in the group]”* (Trumpet player 2. rehearsal 1).

Data from observations and recordings of rehearsals also indicated that there were collective evaluation processes by participants regarding the group’s performance:

- Regarding rhythmic precision: *“(...) it was a bit off, right? [Expressions of agreement in the group]”* (Trumpet player 1, rehearsal 1).
- About technical-instrumental variables: *“[It’s difficult] to measure this breathing...”* (Trumpet player 2, rehearsal2).
- About nuances of dynamics: *“Is it possible for you [trombonist] to reduce it a little less?”* (Horn player; rehearsal 2).
- About agogic inflections: *“(...) here it’s just a ritenuto and continues. We’re doing a fermata...”* (Trumpet player 1; rehearsal 2).

As a result of the evaluation processes, self-reactive initiatives were observed. In this sense, it is possible to highlight: the conscious and deliberate repetition of specific passages, the study in parts guided by the analysis of structuring aspects of the music and the collective resolution of interpretative and technical-instrumental problems, based on dialog and experimentation with strategies.

Summarizing, the data presented here highlights the occurrence of cognitive processes, affective and motivational undertakings pertinent to the processes of metacognitive regulation, resulting from planning (outlining goals and activities), monitoring (metacognitive experiences and knowledge), evaluation and self-reaction (evaluative inferences and self-reactive initiatives) in musical practice and learning of the investigated chamber ensemble.

### 3.2 Second study: beginner violin students

During the lesson observations, it was found that the teacher acted in a way that encouraged the students to learn reflectively and autonomously, leading them to develop their metacognitive processes. The interviews served to confirm how the three students developed their metacognitive processes while studying the violin in their individual musical practices. The students’ study practice was based on the instructions given by the teacher weekly. Participant 1 (P1) spent an average of 6 hours a week studying the violin. P1 was in the eighth term of his computer science course. P2 spent an average of 1 hour a week studying the violin, and was in the sixth term of his bachelor’s degree in music production; P3 was in the seventh term of his computer science degree and spent an average of 8 hours a week studying. It was possible to observe, therefore, that the beginning students dedicated fewer hours to studying than the professional musicians in case 1 (who had more than 8 h of weekly practice). In the first part of the interview, about students’ motivation for learning the violin, it was possible to identify that they chose to learn the instrument mainly through intrinsic motivation. P1 commented: “*I like playing the violin*,” “*I want to learn new skills*,” “*It’s fun*.” P2 and P3 also stated that they did not feel pressured to study the violin, as they were learning because they liked playing the instrument.

To analyze the metacognitive processes, the first group of questions focused on the *planning* stage. Participants were asked about planning their studies. Initially, they mainly highlighted planning regarding the place and time of study. P1 and P3 reported that they studied at home, with the concern of studying *“inside the room, so as not to disturb the neighbors. No later than 8 or 9 pm.”* Both also said that the ideal would be to study inside the classroom where the lessons take place, because *“it’s a large place, with good acoustics and quiet, and it does not disturb people with the noise,”* said P3. P2, who attended the place where the violin lessons were held, said that he studied in the same place as the lessons, but that *“any place that is quiet helps; it does not matter where it is, because if you study in a concentrated way, what happens around you does not get in the way.”* When asked if the place where they studied favored their development or if they would like to be able to study elsewhere, P2 and P3 replied that *“it would be better to study in the room where the classes take place, but for convenience I study at home.”* P1 said he did not know, because for him the only ideal place to study would be *“where there was someone else to correct my mistakes!.”* At this stage of planning, in addition to identifying where they planned to practice, participants also indicated that they knew what they planned to practice, highlighting technical exercises and repertoire pieces worked on that week by the teacher.

Regarding the management of time to practice, still in the planning stage, P2 said: *“I cannot organize my study time in this course. In other classes, I even try to be more organized, but without much success. I have serious organizational problems.”* P1 and P3 said that they were able to plan their time to practice. P1 said that “*when I manage to plan my day, I fit in 20 or 30 min a day to practice the violin*” and P3 explained that during the week, she was able to practice in the morning, as the degree course she was taking had afternoon and evening classes. According to Benton (2014), among the metacognitive skills advocated by several authors, task planning is an important factor. Thus, from the data collected, it was possible to observe that building a favorable environment for studying the violin and organizing practice time, even with difficulties, were processes sought by students.

The *monitoring* stage was addressed in the interview, especially focusing on the content covered in violin classes. When asked if they were able to understand and perform the content covered in class, P2 and P3 said that the repertoire covered was easy, as they already knew some of the songs. However, they argued that they found it difficult the way the teacher asked them to play the pieces, with the technical specifications he demanded and the sound he required (see Table 3).

**Table 3:** Study monitoring.

Interviewed	Interview data
P1	*In the first semester, I found it very difficult, but I think it’s because I did not have much preparation. I know that I do not have the knowledge to read scores fluently like other students, and that’s why I was a little behind in class. Then last semester it was very difficult. But it’s my fault for not preparing for classes in advance.* *But this semester I prepare more in advance. I see in advance what the subjects are, what the songs are, and I make notes on the scores, trying to play a little too to learn the theoretical part better, with a greater emphasis on the score (score reading).*
P2	*(...) the score is easy. It’s just the technical issues that are difficult. If I just played the notes, the way I always did, it would be very easy!* *(...) trying to get a constant sound, throughout the entire arc, was very challenging. But I liked it, because I was able to do it without having much time to study!* *(...) playing any way is easy, the problem is playing correctly, the way the teacher asks.*
P3	*(...) playing any way is easy, the problem is playing correctly, the way the teacher asks.*

For Flavell (1979) *monitoring* includes a variety of actions and interactions from four classes of phenomena: (a) metacognitive knowledge, (b) metacognitive experiences, (c) objectives (or tasks) and (d) actions (or strategies). In addition, according to Flavell (1979) and Ribeiro (2003) metacognitive experiences occur in situations that stimulate attention and include conscious thoughts. In fact, these reflective processes were observed in the participants’ responses, directly or, at times, implicitly. In this sense, Flavell (1979) indicates that these situations that allow reflection on thoughts and feelings and the quality control of metacognitive experiences, can cause elements of cognitive knowledge to be added, deleted or revised.

With regard to evaluating their performance in the practical sections, participants indicated that they should focus more of their efforts on improving the technical aspects presented by the teacher (see Table 4).

**Table 4:** Performance evaluation.

Interviewed	Interview data
P1	*“During the practice, I do not know what I could change... I do not know if I should put in more technique, more repetitions, and how these repetitions should be done. But basically to have more consistency in the study.”*
P2	*“I was so anxious trying to hold the bow straight, to position the violin in the right place, to get the fingers of the left hand right, and to maintain a correct posture, that I often ended up getting lost! And then with sheet music, it got worse! I did not know where to look anymore... I’d look at the score and forget everything else... I’d look at everything else and forget the score. But when I memorized the music, and when the teacher told me to focus on the sound being produced, I felt calmer. I knew that if there was a sound I did not like, there was something wrong. And I learned to find out what was wrong just by listening!”* *“I would need to study at least 30 min a day to be able to do it the way the teacher asks.”*
P3	*“So I think I should do the exercises a bit more.”* *“I feel like here I’m learning from much better quality.”*

Benton (2014) reports that many beginner music students of school age simply practice by playing entire pieces repetitively, without detecting errors and stopping to correct them, drawing attention to an erroneous way of practicing, as it generates a counterproductive effect since students reinforce errors. In our study we also found this situation in P2’s interview. In this sense, deliberate practice should be reinforced in learning, because repetition without reflection can generate errors and inappropriate techniques, instead of correcting them. This only highlights the importance of conscious deliberate practice, using all the metacognitive factors related to monitoring and evaluating cognition, bringing correction strategies to verify the best way to change strategies that have not had an effect, which are advocated by Schraw and Dennison (1994). According to Benton (2014), when an individual uses metacognition, their object of thought is the personal act of knowing or the intellectual process of obtaining knowledge.

The final questions in the interview were about seeking help to practice and to improve the performance. The participants were asked if they sought help from their teacher and peers (peer review) when they had any doubts. Everyone answered positively highlighting that they felt free to ask their teacher any questions they had. This was confirmed during class observations. According to them, the freedom to talk to the teacher had important consequences for learning. P3 said: *“I feel that here I’m learning with much more quality. The teacher wants us to learn properly. The teacher does not come here just to keep to the timetable and get paid at the end of the month. He wants us to learn!.”* The participants also confirmed that they used to exchange information with colleagues. P1 commented that he talked to other colleagues, often by text message on his cell phone: *“Sometimes I even send them a photo of the score so they can help me read the notes.”* This way of practicing, exchanging information with colleagues, using technological resources, such as cell phone photos, exchanging messages, therefore, is a specificity found in this study on the way in which beginning university students practiced. These specificities were not found in the data from other studies that we reviewed on the metacognitive processes of young instrumentalists.

Finally, when evaluating their own performance in comparison with each other’s, all participants stated that they noticed individual differences, highlighting that some had faster development in one skill (e.g., tuning), while others developed faster in other skills. (e.g., reading, interpretation, etc.). Concina (2019) states that the relationship between metacognition and expertise is characterized by a gradual process of transfer of learning, where students learn metacognitive strategies within a particular cognitive domain, and then go on to transfer their metacognitive skills to other areas of learning. According to the author, educators can play a key role in promoting this process, encouraging students to apply what they have learned to other areas of learning.

## 4 Discussion

By analyzing the two case studies, carried out with professional musicians and beginner college musicians, it was possible to verify that, even at different levels of expertise, metacognitive processes were present in the participants’ musical practices and were declared through the musicians’ reflective processes.

About metacognitive processes, in both cases (beginners and experts), it was possible to verify that study organization was a component present of the *planning* process in metacognitive regulation. However, it was noticeable that, for beginner musicians, organizing activities and managing study time was a significantly more difficult compared to the group of professional musicians. The reported difficulty was often related to external factors, which participants often could not control.

Regarding the *monitoring* and *evaluation* processes, it was possible to verify that both the beginning and the professional musicians used declarative knowledge (in the form of self-knowledge), conditional knowledge (awareness of the demands of the tasks) and procedural knowledge (problem-solving strategies) to carry out and reflect on their musical activity. This result follows the direction of the study by Power and Powell (2018), who, when carrying out their study with children and teenagers, found that increasing awareness of metacognitive processes impacts on improving the quality of learning processes for beginner instrumentalists. In our study, when working with adults, we could question whether beginning adults, when developing metacognitive skills in other domains, could transfer these skills to instrumental learning. This issue, however, could be a topic for further study.

Overall, this research is in line with Hallam (2001)’s study, which highlighted the difference in the metacognitive processes of beginner students and professional musicians. According to the author, professionals demonstrate advanced metacognitive skills in relation to the construction of performance, covering technical and interpretative elements, as well as issues related to learning, such as concentration, planning, monitoring and evaluation. The author also indicated in her study that, for beginner students, “there was a complex relationship between the development of knowledge and the use of planning strategies” (p. 27).

The results of the two studies we carried out also demonstrated that there is a difference between the metacognitive skills of (adult) beginners and expert musicians. Expert musicians have greater self-reflection about their performance and how to improve the quality of their performances. This result follows the direction of the study by Concina (2019), indicating that the student’s level of musical experience has an impact on metacognitive skills. According to the author, experienced musicians exhibit more developed metacognitive attitudes and behaviors, highlighting that advanced or professional students can select the most appropriate strategies, understand the level of difficulty and possible challenges of a task, monitor their performance and allocate the amount of time needed to solve each challenge, optimizing their efforts in the learning activity.

Data from the two case studies led us to understand that the development of metacognitive skills allows the instrumentalist to follow their own musical path autonomously. This finding follows the direction of the study by Hallam (2001). The author indicated that the musician must develop considerable metacognitive skills to be able to recognize the nature and requirements of a task in order to identify particular difficulties and have knowledge of a series of strategies to deal with these challenges. Furthermore, the author points out that it is necessary to know which strategy is appropriate to deal with each task, as well as monitor progress and, if there is unsatisfactory progress, use alternative strategies to finally evaluate learning results and take necessary measures to improve performance. Our results, therefore, add to the results already achieved by Hallam, bringing as a genuine contribution the look at a different geographic and sociocultural context and a different population, which included adult beginner violin students and professional musicians from a chamber group, offering a new perspective to studies on metacognition and musical practice.

## 5 Conclusion

We observed that in both case studies time management was a component of the *planning* process in metacognitive regulation, however for the beginner violinists this was a more complex task compared to the group of professional musicians. In relation to learning *monitoring* processes, it was possible to verify that both adult beginner musicians and professional musicians used declarative, conditional and procedural knowledge to carry out and reflect on their musical practices, however, professional musicians demonstrated that they had greater reflective capacity, a situation resulting from their musician/professional experiences. Regarding the *evaluation* process, it was possible to verify that professional musicians were able to judge their own behavior in relation to musical progress and the relevance of the resources and strategies used in study practices. Beginner musicians judged their behavior, but did not always know how to evaluate the effectiveness of the strategies used. These results have implications for both the teaching process and the musical study process. In this sense, it is observed that reflective thinking and the use of strategies must accompany individual and collective musical practices for improvements in music studies to occur, since as Mitsea and Drigas (2019) indicate there is a significant co-occurrence between high-level cognitive functions (such as reasoning, critical thinking and problem solving) and the use of metacognitive strategies. We therefore reinforce the idea that metacognition is a relevant process to guide the practice of instrumentalists (in individual and collective contexts), as it involves conscious, reflective and autonomous musical development.

## Data availability statement

The original contributions presented in the study are included in the article/supplementary material, further inquiries can be directed to the corresponding author.

## Ethics statement

The studies involving humans were approved by Ethics Committee of the Health Sciences Sector of the Federal University of Paraná - SCS/UFPR. Number: 41065320.6.0000.0102. The studies were conducted in accordance with the local legislation and institutional requirements. The participants provided their written informed consent to participate in this study. Written informed consent was obtained from the individual(s) for the publication of any potentially identifiable images or data included in this article.

## Author contributions

RCA: Writing – original draft, Writing – review & editing. RSF: Writing – original draft, Writing – review & editing. FDDV: Writing – original draft, Writing – review & editing.

## Footnotes

^1^See the interview questions in: Flávio Denis Dias Veloso. “A prática e a aprendizagem da performance musical em grupos de câmara: uma investigação sob a perspectiva sociocognitiva.” (PhD diss., Veloso, 2022).

^2^See the interview questions in: Rafael Stefanichen Ferronato. “Um estudo longitudinal sobre autodeterminação e processos metacognitivos na aprendizagem do violino.” (PhD diss., Veloso, 2022).
